# Supplementary material for: Low credibility URL sharing on Twitter during reporting linking rare blood clots with the Oxford/AstraZeneca COVID-19 vaccine
Source: PLoS One. 2024 Jan 19;19(1):e0296444. doi: 10.1371/journal.pone.0296444 (PMC10798519; doi:10.1371/journal.pone.0296444)
Supplement: S1 Table — (DOCX) [file pone.0296444.s001.docx]

**S1 Table. Keywords used in the Twitter API to create the COVID Corpus.**

| **Language** | **Keywords** |
| --- | --- |
| English | coronavirus, cov19, covid, covid19, Covid-19, corona, SARS-CoV-2, coronavirus, corona virus, pandemic, epidemic, outbreak, infection, pneumonia, ventilators, swab, ICU, PPE, NHS, antibody, 111, Mask, Gloves, FFP-3, Diarrhea, Diarrhoea, #codiv-19, #covid, #covid_19, #COVID2019, #COVID19, #StayAtHome, #lockdown, #lockdownUK, #Covid19Impact, #NHSNightingale, #ProtectNHS, #StayHomeSaveLives, #QuarantineLife, #HerdImmunity, #21DaysChallenge, #Homeschooling2020, #WashYourHands, #StaySafe, Vaccination, vaccine, jab, RNA vaccine, anti-vac, anti-vax, Pfizer, BioNTech, Oxford, #antivax, antivaxxers, #Covidvaccine, #Pfizer, #Pfizervaccine, #BioNTech, #vaccinecovid, #vaccinecovid |
| Spanish | pandemia, epidemia, brote, infeccioso, infección, respiratorio, neumonía, neumonia, infeccioso, ventiladores, frotis, UCI, EPI, anticuerpo, #QuedateEnCasa, #QuedateEnLaCasa, #QuedateEnTuCasa, #VidaEnCuarentena, #LavateLasManos, #covid19ecuador, #covid19mx, #covid19chile |
| Portuguese | corona vírus, pandemia, epidemia, surto, infecção, respiratória, pneumonia, infecção, infeccioso, infecciosa, ventiladores, respiradores, cotonete, esfregão, esfregaço, UTI, EPI, equipamento de proteção individual, equipamentos de proteção individual, SUS, Sistema Único de Saúde, Sistema de Saúde, Saúde Pública, anticorpo, 192, #FiqueEmCasa, #IsolamentoSocial, #DistanciaSalva, #ImpactoCovid19, #NHSNightingale, #ProtejaOSUS, #FicarEmCasaSalvaVidas, #VidaEmQuarentena, #EstudeEmCasa2020, #LaveAsMaos, #FiqueSeguro |
| German | Pandemie, Epidemie, Ausbruch, Atemwegsinfektion, Lungenentzündung, Pneumonie, infektiös, Beatmungsgerät, Abstrich, ITS, Intensivstation, persönliche Schutzausrüstung (PSA), oder Schutzkleidung, Krankenhaus, Antikörper, 110, 112, 19222, Atemschutzmasken, Handschuhe, FFP-3, Durchfall, #BleibzuHause, #Ausgangssperre, #AusgangssperreDeutschland, #Covid19impact, #Covid19Auswirkung, #zuhauseBleibenLebenRetten, #LebenInQuarantäne, #Herdimmunität, #21DayChallenge, #21TageHerausvorderrung, #Homeschooling2020, #Haussunterricht2020, #häuslicherUnterricht2020, #WaschDeineHände, #HändeWaschen, #StaySafe, #PassAufDichAuf |
| French | Epidémie, épidémie, infection, respiratoire, pneumonie, infectieux, infectieuse, contagieux, respirateur, prélèvement, Unité de soins intensifs (USI), équipement de protection individuelle, (EPI), DGS, Direction Générale de la Santé, anticorps, 15, 112, #Arrêt, #distanciation sociale, #lockdownFrance" |
| Arabic | فايروس, كورونا, سارس, وباء, وبائي, انتشار, التهاب, رئوي, العدوى, العناية, المركزة, الصحة, مضاد, 937, كوفيد#, كوفيد-19#, خلك_بالبيت#, الحجر#, منع_التجول#, فعاليات_الحجر_المنزلي#, أبطال_الصحة# |
| Chinese | 新冠, 病毒, 抗疫, 病例, 感染, 流行病, 大流行, 传染病, 疫情, 呼吸道感染, 肺炎, 传染, 呼吸机, 核检测, 咽拭子, 防护, 医疗, 疫苗, 120, #新冠病毒 |
