# Supplementary material for: Low credibility URL sharing on Twitter during reporting linking rare blood clots with the Oxford/AstraZeneca COVID-19 vaccine
Source: PLoS One. 2024 Jan 19;19(1):e0296444. doi: 10.1371/journal.pone.0296444 (PMC10798519; doi:10.1371/journal.pone.0296444)
Supplement: S1 Appendix — (DOCX) [file pone.0296444.s002.docx]

**S2 Appendix. Keywords used to create the AZ Corpus.**

AstraZeneca, Astra Zeneca, AZ, AZD1222, Vaxzevria, Oxford vaccine.
