# Supplementary material for: Low credibility URL sharing on Twitter during reporting linking rare blood clots with the Oxford/AstraZeneca COVID-19 vaccine
Source: PLoS One. 2024 Jan 19;19(1):e0296444. doi: 10.1371/journal.pone.0296444 (PMC10798519; doi:10.1371/journal.pone.0296444)
Supplement: Attachment — Submitted filename: recommendation.pdf [file pone.0296444.s057.pdf]

Report on Manuscript PONE-D-23-16069

Overview

The manuscript *Identifying how the diffusion of low-credibility COVID-19-related information on Twitter reacts to reports linking rare blood clots with the Oxford/AstraZeneca COVID-19 vaccine* analyses the diffusion of low-credibility content about the Oxford/AstraZeneca COVID-19 in the aftermath of concerns raised on the possible link between that vaccine and medical complications like blood clots.

The authors used social network analysis tools like the library NetworkX of the Python programming language, as well as time series of contents posted on Twitter on a time-span of three weeks around the peak days of social engagement on the topic.

The manuscript is well-written, well-presented, and the research conducted within it is interesting and, to my knowledge, new. For these reasons, I recommend it for publication, even if there are some points that I would like to be clarified or improved.

Questions

Line 19 Regarding the keyword list to create the corpus in the Annex A, why did not you consider also keywords in Italian? Italy was one of the most affected countries by COVID-19 in Europe and vaccines were a major political issue, daily discussed on the main media and information bodies.

Figures (general) Figure quality (i.e., resolution) can be improved, except maybe Figures 3, 5, 9, 12 and 13.

Figures 4, 6, 7 I find the light blue background not of help, readability-wise, especially if paired with the colors employed for the lines.

Table 6 "Low credibility" in the header on the left is partially unreadable.

Bibliography I think the authors can mention the paper

> Agosti F, Toffolutti V, Cavalli N, Nivakoski S, Mascherini M, Aassve A (2022) Information and vaccine hesitancy: Evidence from the early stage of the vaccine roll-out in 28 European countries. PLoS ONE 17(9): e0273555.
>
> `https://doi.org/10.1371/journal.pone.0273555`

among their references.
