# Supplementary material for: Low credibility URL sharing on Twitter during reporting linking rare blood clots with the Oxford/AstraZeneca COVID-19 vaccine
Source: PLoS One. 2024 Jan 19;19(1):e0296444. doi: 10.1371/journal.pone.0296444 (PMC10798519; doi:10.1371/journal.pone.0296444)
Supplement: Attachment — Submitted filename: Response to Reviewers.docx [file pone.0296444.s058.docx]

| **Comments** | **Reply** |
| --- | --- |
| Journal Requirements | |
| Please ensure that your manuscript meets PLOS ONE's style requirements, including those for file naming. | The following updates have been made:   - Cover page updated - Level 1, 2, and 3 heading text sizes updated - One heading updated to sentence case - Figure citations updated - Figure captions updated - Where not already the case, figure captions moved to directly after the paragraph in which they are first cited - Table captions updated - Where not already the case, table captions moved to directly after the paragraph in which they are first cited - Reference citations updated to square brackets - Referencing style updated to Vancouver - Supporting Information citations updated - Supporting Information removed from manuscript and uploaded separately as individual files. - Supporting Information section within manuscript updated to solely include the cations and titles of the supporting information |
| In your Methods section, please include additional information about your dataset and ensure that you have included a statement specifying whether the collection and analysis method complied with the terms and conditions for the source of the data. | Additional details provided about the data collection. A statement has been added to confirm that the collection and analysis method complied with the terms and conditions for the source of the data. |
| Please upload a new copy of Figures 5a and 5b as the detail is not clear. | Updated as requested. |
| Please include captions for your Supporting Information files at the end of your manuscript, and update any in-text citations to match accordingly. | Updated as requested. |
| Please review your reference list to ensure that it is complete and correct. If you have cited papers that have been retracted, please include the rationale for doing so in the manuscript text, or remove these references and replace them with relevant current references. Any changes to the reference list should be mentioned in the rebuttal letter that accompanies your revised manuscript. If you need to cite a retracted article, indicate the article’s retracted status in the References list and also include a citation and full reference for the retraction notice. | [Check on extra paper recommended by reviewer 2].  Missing reference to Ahmed et al. added.  Additional references added to Durazzi et al., as suggested by Reviewer #1, and  Reference list reviewed. No corrections made. None of the cited papers have been retracted. |
| Reviewer #1 Comments | |
| Title: I think that a shorter title could help to understand what the study is about. | Title updated to “Low credibility URL sharing on Twitter during reporting linking rare blood clots with the Oxford/AstraZeneca COVID-19 Vaccine” |
| Line 2: Readers in the future may be not so familiar with the term COVID-19 alone. Authors could introduce the fact that there has been a pandemic, or at least write “The COVID-19 pandemic has occurred etc”. | Additional context around the COVID-19 pandemic added in an opening sentence. |
| Line 50: I think it is important to check and report whether these websites verify sources in all the different languages that you are collecting. Otherwise, some low-credibility sources may be neglected only because the Iffy dataset did not do any verification at all for websites in some specific languages. In this, case this should be properly enunciated in the limitations of the study. | The Iffy+ list covers 19 languages, including all seven of the languages that we were collecting against. This has been noted in the methods section under the “Data collection and pre-processing”. |
| Line 83 (and elsewhere): "network" and "graph" synonyms, thus the usage of "network graph" is improper. I would recommend using only the term "network", consistently along the paper. | 13 instances of “network graph” updated to “network”. |
| Line 89: Authors should specify here which is the direction of the edge | Direction (from the originator of the tweet to the user who retweeted it) added. |
| Line 201 and Table 2: When an average quantity is shown, also standard deviation could be shown alongside (e.g. average +- std), to help understand when a quantity is greater or lower than another in the absence of statistical tests. | Standard deviations and statistical tests (Mann Whitney U) added. |
| Line 214: since the authors are measuring the lag until the first retweet only, I am not sure that “speed of diffusion” is the most appropriate term. With “Speed” one usually refers to a quantity per time unit ( e.g. average number of retweets per day, or average potential exposure per day). I would consider other possible names if the authors have other ideas. | Updated from “speed of diffusion” to “time to first retweet”, with other references to speed updated to “time elapsed” where required. |
| Line 253: Authors should mention in the Methods which algorithm was used for network layout (computations of coordinates). The interpretation of proximity in the plot depends on the algorithm chosen. | Details of the visualisation algorithm added under the “Network composition” section of the methods. |
| Line 289: the density of a network can be easily computed. Authors could report a numeric result instead that relying on a figure. | Gephi, the visualisation software used, reports a density of 0.0 for all graphs produced in this research. This is why visual comparison was relied upon. |
| Figure 14-19: for some reason, the pdf file I have been provided does not contain images from 14 onwards. For this reason, I cannot review them for now. | Files have been reuploaded. |
| Line 360: Authors could specify that here "association" is inferred by manually inspecting the user, and it is not an association with preferential sharing of a particular source. | A sentence to this effect has been added. |
| Line 407-409: If I understood well, the correlation is not only with low-credibility, but with AZ-related tweets in general. I would rephrase to say that the correlation is with AZ-related tweets in general, and in particular with tweets containing URLs associated with low-credibility sources. | Updated as suggested. |
| Line 425-428: I am not sure that the authors are providing numerical results supporting this statement (e.g. community detection/clustering), and I don't think the figures alone can support your statement. Therefore, I suggest removing these two sentences from the final discussion, or at least moving them as an additional comment (not a proper result), after the next sentence where you refer to the degree centrality results. Alternatively, authors can perform additional analyses to quantify the variation of clusters. | Sentence order changed as suggested. |
| Line 466: typo: “would be expected TO feature” | Updated as requested. |
| Line 470-471: This sentence is not very clear in English, at least to me. I suggest to rephrase it. | The final two sentences of this paragraph have been updated to make them clearer. |
| Line 502: missing reference to Ahmed et al.. | Citation and reference added. |
| Line 504: Interesting comment. It has also been shown that in the very first months of the pandemic, the international scientific community lost its audience on Twitter, and national political communities became the most prominent authors (Clusters of science and health related Twitter users become more isolated during the COVID-19 pandemic, Sci Rep 2021). Authors dispose of a rich multilingual dataset but do not explicitly use multilingualism to add some richness to the results. I think the authors may add some insights to the mentioned study about the "nationalization" of the debate, by showing the prominent language of each author in Table 6 (e.g. KenRoth (eng) ) and comment appropriately | Reference added to Durazzi et al. which identifies the disproportionate attention received by national elites and political actors by mid-2020.  Language preferences added to Table 6 and added to the results summary in the paragraph above the table. A discussion of these results has been added to the Discussion section on “Influential domains and users”. |
|  | |
| Line 19 Regarding the keyword list to create the corpus in the Annex A, why did not you consider also keywords in Italian? Italy was one of the most affected countries by COVID-19 in Europe and vaccines were a major political issue, daily discussed on the main media and information bodies. | Data collection was initially done in Italian, but firstly data collection began in April 2020 after the first wave in Italy was coming down, and secondly we did not get enough data in Italian. The decision was taken to restrict languages to the most frequent ones. We have added a note to the limitations to reflect this, as well as other languages spoken in countries with an AZ vaccination programme. |
| Figures (general) Figure quality (i.e., resolution) can be improved, except maybe Figures 3, 5, 9, 12 and 13. | Updated versions of the figures have been uploaded. |
| Figures 4, 6, 7 I find the light blue background not of help, readability-wise, especially if paired  with the colors employed for the lines. | Updated plots to remove grey background. |
| Table 6 “Low credibility” in the header on the left is partially unreadable. | Table reformatted. |
| Bibliography I think the authors can mention the paper Agosti F, Toffolutti V, Cavalli N, Nivakoski S, Mascherini M, Aassve A (2022) Information and vaccine hesitancy: Evidence from the early stage of the vaccine roll-out  in 28 European countries. PLoS ONE 17(9): e0273555. <https://doi.org/10.1371/journal.pone.0273555> among their references. | This article has now been referenced in the Introduction. |
